# A Rare Choledochal Cyst in a Child With Abdominal Pain

**DOI:** 10.7759/cureus.82324

**Published:** 2025-04-15

**Authors:** James R Laney, William K Oelsner, Andrew Mims, Samuel Igbinedion

**Affiliations:** 1 Internal Medicine, University of Tennessee Health Science Center College of Medicine - Chattanooga, Chattanooga, USA; 2 Gastroenterology and Hepatology, University of Tennessee Health Science Center College of Medicine - Chattanooga, Chattanooga, USA

**Keywords:** apbj, biliary malignancy, biliary tree anatomical variance, cholangiocarcinoma surveillance, choledochal cysts

## Abstract

Choledochal cysts are rare anatomical anomalies of the biliary tree. These lesions possess a risk for future development of catastrophic malignancy in the form of cholangiocarcinoma if left unaddressed, which is associated with a high mortality rate in cases of malignant transformation. The presenting symptoms of choledochal cysts are often nonspecific. The age of the initial onset of symptoms can vary, and some patients with choledochal cysts do not receive a diagnosis until reaching adulthood. Here, we present a case that serves as an example of early detection of this rare clinical finding for potentially devastating malignancy in a child who presented with nonspecific symptoms.

## Introduction

A choledochal cyst, also referred to as a biliary cyst, is an acquired or congenital anatomical anomaly that leads to the dilation of extrahepatic and intrahepatic components of the biliary tree [1]. Although the precise etiology of choledochal cysts remains unclear, the most widely accepted theory is that these cysts result from an anomalous pancreaticobiliary junction (APBJ), where the pancreatic and biliary ducts join proximal to the sphincter of Oddi [2,3]. The resultant long channel proximal to the sphincter allows for the addition of pancreatic secretions into the common duct, which leads to inflammation, dilation, epithelial damage, fibrosis, and, ultimately, cyst formation [3].

The clinical presentation of choledochal cysts most often features a nonspecific trio of symptoms, including abdominal pain, jaundice, and a palpable abdominal mass [1,4]. The gold standard for diagnosing choledochal cysts is via the imaging modality of magnetic retrograde cholangiopancreatography (MRCP), which has a sensitivity between 90% and 100% [5].

Due to the high risk for future malignancy, choledochal cysts are definitively managed with excision of the cyst and restoration of bile flow via Roux-en-Y hepaticojejunostomy or, less commonly, hepaticoduodenostomy [6]. In this case report, a pediatric patient with nonspecific abdominal symptoms was found to have a Type IA choledochal cyst. This case proves to be clinically relevant, given how it remains a rare anatomical finding with poor outcomes if unaddressed.

## Case presentation

**Figure 1:**
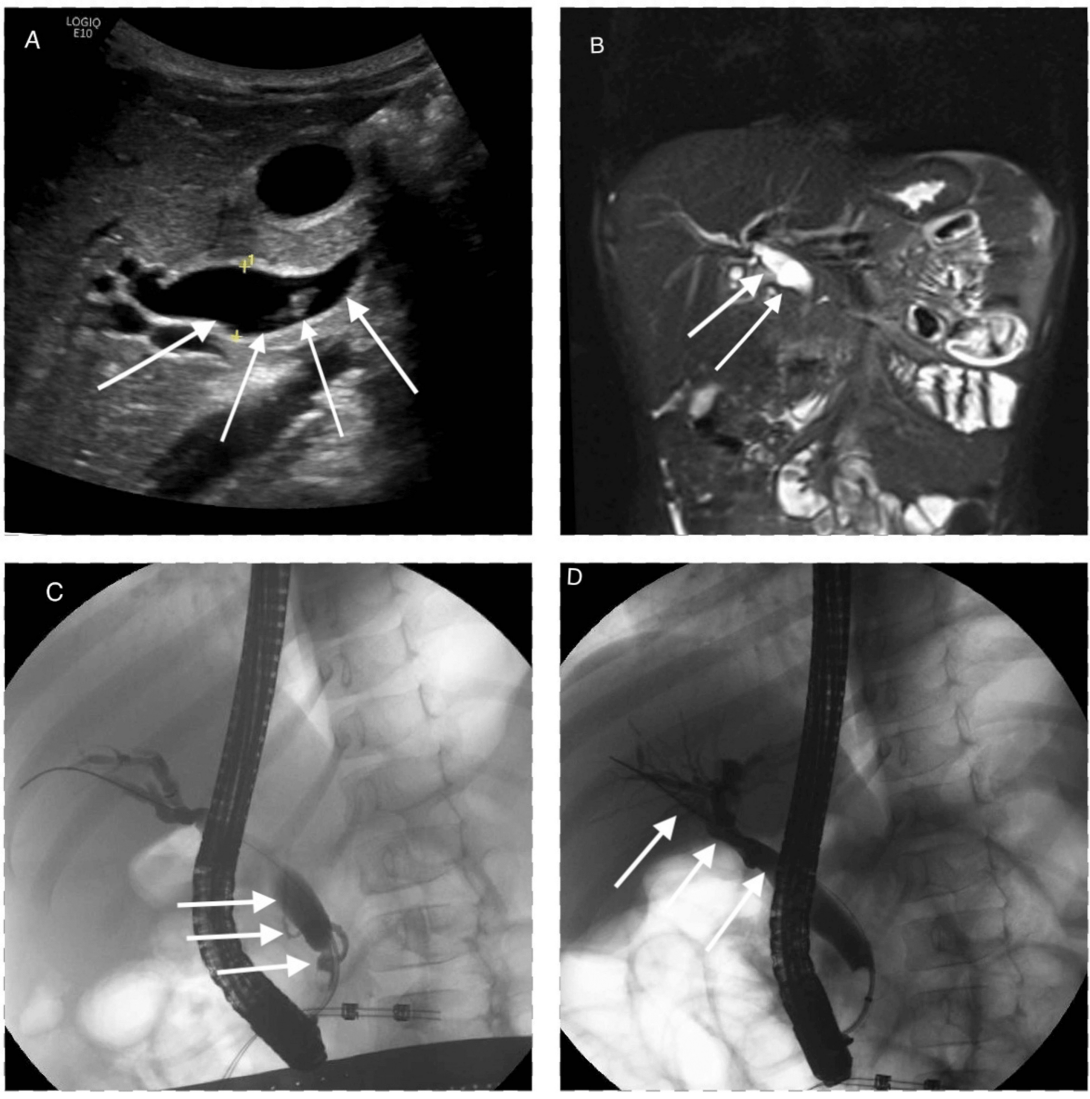
Radiographic imaging of dilated common bile duct, APBJ, and choledochal cyst highlighted by arrows. (A) Ultrasound with evidence of fusiform common bile duct dilation with choledocholithiasis; (B) coronal view of magnetic resonance imaging (MRI) of the abdomen with common bile duct dilation; (C) fluoroscopic imaging during ERCP with evidence of an aberrant pancreatic biliary junction; (D) fluoroscopic imaging during ERCP with identification of Type IA choledochal cyst. APBJ: anomalous pancreaticobiliary junction

A six-year-old female patient who was previously healthy presented to the emergency department following a weeklong course of progressive generalized abdominal pain with vomiting. The initial lab workup was remarkable for an elevated lipase of 3,197 U/L, total bilirubin of 1.4 mg/dL, aspartate aminotransferase (AST) of 317 U/L, alanine aminotransferase (ALT) of 682 U/L, white blood cell (WBC) count of 11.2 × 10^3^ cells/mm^3^, and hemoglobin (Hgb) of 14.2 g/dL (Table 1). An ultrasound of the right upper quadrant showed diffuse enlargement of the pancreas with peripancreatic edema, cholelithiasis, and biliary dilation with the common bile duct measuring up to 11 mm, for which gastroenterology service was consulted for further evaluation with endoscopic retrograde cholangiopancreatography (ERCP) (Figure 1A). The ERCP procedure demonstrated evidence of choledocholithiasis.

**Table 1:** Initial laboratory results

Parameter	Result	Reference Range
Lipase (U/L)	3197	0–160
Total bilirubin (mg/dL)	1.4	0.2–1.3
Aspartate aminotransferase (U/L)	317	15–50
Alanine aminotransferase (U/L)	682	5–25
White blood cells (×10^3^ cells/mm^3^)	11.2	5–19
Hemoglobin (g/dL)	14.2	9.5–14

Complete stone removal was accomplished by biliary sphincterotomy and balloon extraction. Upon review of cholangiogram imaging, there was a concern for an APBJ (Figure 1C). Further workup with MRCP was pursued, which confirmed the presence of APBJ with a long common channel measuring 10 mm. Dilation of the extrahepatic common bile duct with milder intrahepatic ductal dilation evident on both fluoroscopic images from ERCP (Figure 1D) and on MRCP (Figure 1B) confirmed the suspected diagnosis of a Type IA choledochal cyst in the setting of APBJ. Following this diagnosis, there was a change in management as the pediatric surgery team halted plans for cholecystectomy. The hepatobiliary surgery team was then consulted, and the patient was discharged with outpatient plans for a hepaticojejunostomy.

## Discussion

Choledochal cysts remain an extremely rare finding in children in the United States, with an incidence of one in 150,000 and a 1:4 male-to-female rate ratio [4,7]. Choledochal cysts are classically categorized into five classification groups, with Type I involving dilation of the common bile duct [8]. Type I represents the majority of cases, comprising between 50% and 80% of all choledochal cysts [8,9]. Type I can be further subdivided into IA, IB, or IC, with IA exhibiting cystic dilation of the extrahepatic biliary tree in the presence of APBJ [8]. These choledochal malformations have been shown to carry around a 10% risk for future development of biliary malignancy [10,11]. The proposed theory for the development of malignancy appears to vary based on the subtype of choledochal cyst, but for Type I, the leading theory is that the prolonged exposure to reflux containing pancreatic secretions is responsible for malignant transformation of the biliary epithelium [12,13].

The clinical presentation of choledochal cysts classically features a triad of nonspecific symptoms of jaundice, abdominal pain, and palpable abdominal mass, yet this clinical presentation is only observed in about 17% of cases [14]. Additionally, about two-thirds of choledochal cysts are diagnosed before age 10, following the presentation of clinical symptoms. Additionally, as many as 15% of diagnoses are made incidentally in adulthood, as choledochal cysts in these instances remain asymptomatic [15]. The wide spectrum of presentation of these cysts, from nonspecific findings on initial presentation to incidental diagnosis due to an asymptomatic disease process combined with an extremely poor prognosis from potential development of downstream malignancy, makes early diagnosis, as well as early management with surgical excision, of choledochal cysts paramount.

Outcomes following comprehensive excision of choledochal cysts have demonstrated excellent prognosis with a five-year survival rate of 90% [16]. Despite a better prognosis with surgery, around 10% of all patients with choledochal cysts will develop malignancy despite definitive management with surgery [10,11,17]. Of those who develop cancer, the prognosis is very poor, with a five-year survival rate of 5% [11]. With such poor outcomes from those that develop biliary malignancy following resection of cysts, patients will require lifelong monitoring for the development of biliary cancer with serial abdominal ultrasounds, liver function testing, and CA 19-9 testing [18,19].

## Conclusions

In summary, this case report highlights the importance of considering choledochal cysts in the differential diagnosis of pediatric patients presenting with nonspecific abdominal symptoms. Early detection has been demonstrated to prevent severe complications, particularly with the potential for malignant transformation. Given the devastating mortality among those who develop malignancy, even among those who undergo definitive management with surgical excision, timely diagnosis and appropriate management are crucial. This case underscores the need for continued awareness among clinicians and emphasizes the role of imaging, particularly MRCP, in the early and accurate diagnosis of this rare anatomical outlier.
